# Field Investigation of the Bundibugyo Ebola Virus Disease (BDBV) Outbreak in Ituri Province, Democratic Republic of the Congo: Challenges, Strategies, and Priority Actions for Outbreak Control—An Outbreak Investigation Review

**DOI:** 10.3390/idr18050100

**Published:** 2026-09-08

**Authors:** Muambangu Jean Paul Milambo, Christian Ngandu

**Affiliations:** 1 WSU Institute for Clinical Governance and Healthcare Administration, School of Public Health, Walter Sisulu University, Mthatha 5100, South Africa; 2 Ministry of Public Health, Hygiene and Social Security, Institut National de Santé Publique (INSP), Kinshasa P.O. Box 11850, Democratic Republic of the Congo

**Keywords:** Bundibugyo Ebola virus disease, *Bundibugyo ebolavirus*, Democratic Republic of the Congo, Ituri, outbreak investigation, surveillance, laboratory systems, infection prevention and control, risk communication, infodemic management, community engagement, public health emergency

## Abstract

Background: The 2026 outbreak of Bundibugyo Ebola virus disease (BDBV) in eastern Democratic Republic of the Congo (DRC), centered in Ituri Province, represents the largest documented outbreak caused by *Bundibugyo ebolavirus* since its discovery in Uganda in 2007. The outbreak evolved within a complex humanitarian setting characterized by armed conflict, population displacement, mining-related migration, weak health systems, extensive population mobility, and an infodemic environment marked by misinformation and reduced public trust. We conducted a field investigation to assess epidemiological, operational, laboratory, infection prevention and control (IPC), community engagement, risk communication, and infodemic management challenges and identify priority interventions to strengthen outbreak control. Methods: A rapid field assessment was conducted between 12–15 June 2026 in Bunia, Rwampara Health Zone, and the Ituri Provincial Public Health Laboratory. Data were collected through direct observation, review of surveillance and laboratory reports, health facility assessments, stakeholder interviews, and analysis of outbreak response indicators. Epidemiological trends, surveillance performance, laboratory capacity, clinical care, IPC activities, logistics, risk communication, community engagement, and infodemic management approaches were evaluated. Results: As of 12 July 2026, the outbreak had resulted in 1926 laboratory-confirmed cases and 702 deaths, corresponding to an overall case fatality rate (CFR) of 36.4% across affected provinces. Ituri Province remained the epicenter, accounting for 90.8% of confirmed cases (1705/1877) and 85.5% of reported deaths (577/675). During the preceding 24 h, 53 new confirmed cases and 30 deaths were reported, including 20 community deaths (66.7%), highlighting persistent delays in detection, referral, and access to care. Surveillance systems identified 766 alerts, of which 678 (88.5%) were investigated, resulting in 235 suspected cases. Contact tracing remained a major challenge, with only 64.4% (4171/6475) of registered contacts successfully followed, below the recommended ≥95% target. Laboratory activities included testing of 137 specimens, with 29 positive results and an overall positivity rate of 21.2%. Decentralized molecular diagnostic platforms improved access to testing; however, data inconsistencies, delayed investigations, and gaps in outcome classification affected response monitoring. Major operational challenges included limited treatment capacity, high occupancy of Ebola treatment centres, shortages of trained personnel and IPC supplies, insecurity affecting response teams, and insufficient preparedness in newly affected areas. Community resistance, attacks on burial teams, detention of frontline responders, misinformation, and rumors contributed to delayed care-seeking, reduced acceptance of public health measures, and incomplete cooperation with contact tracing. Risk communication and community engagement efforts were constrained by limited outreach capacity, language barriers, low trust, and inadequate systems for rumor detection and infodemic response. Conclusions: The ongoing BDBV outbreak in eastern DRC demonstrates the difficulty of controlling Ebola transmission in conflict-affected and socially complex settings. Sustained transmission, community deaths, geographic expansion, and operational constraints highlight the urgent need to strengthen surveillance, contact tracing, laboratory systems, IPC capacity, clinical care, and integrated risk communication and infodemic management strategies. Building trust through community-centered approaches, proactive misinformation management, and engagement of trusted local actors will be essential to accelerate outbreak containment and strengthen preparedness across the Great Lakes region.

## 1. Background

Ebola virus disease (EVD) remains one of the most consequential emerging infectious diseases globally because of its high case-fatality rates, potential for rapid amplification within communities and healthcare settings, and profound socioeconomic consequences during large-scale outbreaks. Since the first recognized outbreaks in 1976 in the Democratic Republic of the Congo (DRC) and Sudan, repeated epidemics have highlighted persistent vulnerabilities in surveillance systems, outbreak preparedness, infection prevention and control (IPC), and health-system resilience across sub-Saharan Africa [1,2,3]. The unprecedented West African epidemic of 2014–2016, which resulted in more than 28,000 cases and over 11,000 deaths, demonstrated that Ebola outbreaks can rapidly evolve from localized events into international public health emergencies when early detection and response mechanisms are inadequate [2,4].

Among the five recognized ebolavirus species, *Bundibugyo ebolavirus* (BDBV) remains comparatively understudied despite its substantial epidemic potential. First identified during an outbreak in western Uganda in 2007, BDBV has been associated with significant morbidity and mortality, with reported case-fatality rates ranging between 25% and 50% [5]. Unlike Zaire ebolavirus, for which licensed vaccines and therapeutics are available, no approved vaccine or targeted treatment currently exists for *Bundibugyo ebolavirus*. Consequently, outbreak control relies heavily on rapid case detection, isolation, contact tracing, community engagement, IPC measures, and supportive clinical care, making operational preparedness particularly important in affected settings [5].

The African continent continues to experience recurrent Ebola outbreaks because of a complex interplay of ecological, demographic, and health-system factors. Expanding human encroachment into forest ecosystems, frequent human–animal interactions, increasing population mobility, porous international borders, and weak public health infrastructure collectively facilitate both zoonotic spillover and sustained human-to-human transmission [6,7]. Countries in Central and East Africa remain especially vulnerable because many communities reside in remote areas with limited access to healthcare services and laboratory diagnostics, while regional population movement creates opportunities for rapid cross-border spread once outbreaks emerge [6].

The Democratic Republic of the Congo occupies a unique position in the global epidemiology of Ebola. Since the landmark Yambuku outbreak in 1976, the country has experienced more Ebola outbreaks than any other nation worldwide, including major epidemics in Kikwit, Isiro, Equateur Province, and the North Kivu–Ituri outbreak of 2018–2020 [8,9]. These repeated epidemics have generated substantial experience in outbreak response but have also revealed persistent structural weaknesses, including limited healthcare capacity, delayed detection of transmission chains, community mistrust, and challenges associated with delivering interventions in insecure environments. The recurrence of Ebola outbreaks in DRC underscores the need for continuous strengthening of preparedness systems beyond emergency response periods [8,9].

The current BDBV outbreak emerged in a particularly complex regional context involving extensive cross-border interactions within the Great Lakes region. Ituri Province shares a long and highly porous border with Uganda and is connected through commercial, mining, and humanitarian corridors to neighboring countries including South Sudan, Rwanda, Burundi, Tanzania, and Kenya. Uganda has itself experienced multiple Ebola outbreaks, including the first documented Bundibugyo outbreak in 2007 and subsequent Sudan ebolavirus epidemics [10]. The occurrence of imported BDBV cases during the current epidemic highlights the regional dimensions of outbreak control and emphasizes the importance of coordinated surveillance, information sharing, and preparedness measures across national borders.

A defining characteristic of the 2026 outbreak is its occurrence within an environment affected by chronic insecurity, population displacement, and resource extraction activities. Eastern DRC continues to experience recurrent violence involving armed groups, including the M23 movement, the Allied Democratic Forces (ADF), CODECO militias, and other local factions. Simultaneously, extensive artisanal and industrial mining operations in Ituri attract highly mobile populations who frequently move between mining sites, trading centers, and neighboring countries. Previous research has shown that conflict and insecurity can directly increase Ebola transmission by disrupting healthcare services, restricting access to affected communities, delaying case detection, and reducing adherence to public health interventions [9,11]. The convergence of epidemic transmission, armed conflict, and mining-related migration therefore presents an unusually complex outbreak-control environment.

Although previous studies have examined Ebola epidemiology in conflict settings, limited evidence exists regarding the operational realities of responding to a large *Bundibugyo ebolavirus* outbreak in an active humanitarian and security crisis. Furthermore, the absence of licensed vaccines and therapeutics for BDBV increases reliance on classical public health interventions, placing greater importance on surveillance systems, laboratory diagnostics, logistics, IPC, and community engagement. Understanding how these response pillars function under conditions of insecurity and constrained resources is essential for identifying implementation gaps and developing practical strategies that can strengthen outbreak control in both DRC and neighboring countries facing similar risks [11,12,13].

The present field investigation was undertaken to generate operational evidence from the outbreak epicenter in Ituri Province and to provide actionable recommendations for response strengthening. Specifically, the investigation aimed to assess epidemiological surveillance, contact tracing, laboratory capacity, healthcare readiness, IPC implementation, logistics, risk communication, and community engagement activities while identifying barriers that may impede outbreak containment. The novelty of this study lies in its integration of epidemiological, laboratory, operational, and socio-behavioral observations from the largest recorded Bundibugyo Ebola outbreak to date, occurring within a setting characterized by armed conflict, population displacement, and intense cross-border mobility. By documenting field-level challenges and priority interventions, this investigation seeks to inform evidence-based strategies for strengthening outbreak preparedness and response in the DRC and across the Great Lakes region [12,13,14].

## 2. Methods

### 2.1. Study Design and Field Investigation Approach

This field investigation was conducted using a rapid outbreak assessment methodology adapted from established outbreak investigation frameworks developed by the Centers for Disease Control and Prevention (CDC), the World Health Organization (WHO), and Africa CDC for public health emergencies and aligned with the International Health Regulations (IHR 2005) core capacities to evaluate surveillance, laboratory, coordination, risk communication, emergency preparedness and response, infection prevention and control, and points of entry in the Democratic Republic of the Congo [15,16,17]. The investigation combined descriptive epidemiology, operational assessment, health-system evaluation, laboratory capacity assessment, and qualitative stakeholder consultations to identify factors influencing transmission dynamics and response effectiveness during the 2026 Bundibugyo Ebola virus disease (BDBV) outbreak in eastern Democratic Republic of the Congo (DRC).

The mission was conducted from 12–15 June 2026 in Ituri Province, the epicenter of the outbreak, with site visits to Bunia, Rwampara Health Zone, Ebola Treatment Centers (ETCs), selected health facilities, community surveillance sites, and the Ituri Provincial Public Health Laboratory. The assessment was undertaken as part of an Africa CDC emergency support mission aimed at evaluating outbreak evolution, identifying operational bottlenecks, and recommending priority interventions for strengthening response activities [12].

### 2.2. Study Setting

The investigation was conducted in Ituri Province, northeastern DRC, which shares international borders with Uganda and South Sudan. The province has an estimated population exceeding 8 million inhabitants and hosts more than one million internally displaced persons. The region is characterized by recurrent armed conflict, population displacement, artisanal mining activities, weak health infrastructure, and frequent cross-border population movement. These contextual factors increase vulnerability to infectious disease outbreaks and complicate outbreak response operations [9,11].

At the time of the investigation, the outbreak had spread across three provinces (Ituri, North Kivu, and South Kivu), affecting 31 health zones. Rwampara Health Zone was selected as a priority assessment site because it represented one of the major transmission hotspots and had reported sustained community transmission.

### 2.3. Data Sources and Data Collection

Multiple data sources were reviewed to obtain a comprehensive understanding of the outbreak situation. Epidemiological data were extracted from daily situation reports, surveillance databases, contact-tracing records, laboratory reports, and health-zone line lists maintained by the Ministry of Health, the National Institute of Public Health (INSP), the National Institute for Biomedical Research (INRB), WHO, and Africa CDC [12].

Field observations were conducted using standardized assessment tools adapted from CDC outbreak investigation guidelines. Direct observations focused on surveillance operations, infection prevention and control (IPC) measures, patient management practices, logistics systems, safe and dignified burial activities, community engagement interventions, and laboratory diagnostic procedures.

Key informant interviews and informal discussions were conducted with provincial health authorities, health-zone management teams, clinicians, laboratory personnel, epidemiologists, surveillance officers, IPC specialists, community engagement teams, and response partners. Information collected focused on operational challenges, workforce capacity, logistics, laboratory performance, community perceptions, and security-related barriers affecting response implementation.

### 2.4. Epidemiological Assessment

The epidemiological assessment aimed to characterize the magnitude, distribution, and transmission dynamics of the outbreak. Variables assessed included confirmed cases, suspected cases, deaths, case-fatality rates, geographic distribution, health-zone involvement, contact-tracing performance, alert management, and trends in transmission. Descriptive analyses were performed using routinely collected surveillance data. Outbreak indicators were analyzed according to standard WHO and CDC Ebola response benchmarks, including contact follow-up rates, alert investigation rates, and timeliness of case detection [16,17,18,19].

### 2.5. Health System and Operational Assessment

An operational assessment was conducted to evaluate the functionality of the outbreak response system. Assessment domains included human resources, logistics, transportation, coordination mechanisms, emergency operations, treatment center capacity, supply-chain management, and resource availability. Attention was given to the availability of trained surveillance personnel, outbreak investigators, IPC specialists, laboratory staff, vehicles, fuel, personal protective equipment (PPE), oxygen supplies, and essential medicines [12].

Health facility assessments were performed at treatment centers and referral hospitals to evaluate patient flow, bed occupancy, clinical management capacity, oxygen availability, staffing levels, and implementation of IPC measures.

### 2.6. Laboratory Assessment

Laboratory capacity was evaluated through site visits to the Ituri Provincial Public Health Laboratory and review of diagnostic workflows. Assessment domains included specimen collection, transport systems, biosafety procedures, molecular testing capacity, quality assurance mechanisms, workforce availability, laboratory infrastructure, supply-chain management, and turnaround times.

Diagnostic platforms evaluated included the RADIONE molecular diagnostic system, RealStar^®^ Ebolavirus RT-PCR platform, and GeneXpert systems. Attention was given to opportunities for decentralization of diagnostic services through point-of-care molecular testing to improve access and reduce delays in confirmation of suspected cases [13].

### 2.7. Community Engagement and Socio-Behavioral Assessment

A rapid qualitative assessment was conducted to identify socio-behavioral factors influencing outbreak response. Discussions with community leaders, healthcare workers, survivors, and response teams explored community perceptions of Ebola, health-seeking behavior, adherence to IPC measures, burial practices, trust in public health authorities, and barriers to acceptance of surveillance and treatment interventions.

Findings were used to identify gaps in risk communication and community engagement (RCCE) activities and to generate recommendations for strengthening community participation in outbreak control [14].

### 2.8. Security Assessment

Given the complex humanitarian environment, a security assessment was integrated into the investigation. Information was collected on incidents affecting response activities, including attacks against healthcare workers, interference with surveillance operations, access constraints, population displacement, and the influence of armed groups and mining activities on outbreak transmission dynamics. Security-related findings were analyzed to assess their impact on surveillance, contact tracing, laboratory operations, and healthcare delivery [11].

### 2.9. Data Analysis

Quantitative data were summarized using descriptive statistics, including frequencies, proportions, rates, and percentages. Epidemiological indicators were analyzed at provincial and health-zone levels. Qualitative findings from interviews and field observations were synthesized thematically to identify recurring operational, laboratory, community, and security challenges affecting outbreak control.

### 2.10. Ethical Considerations

This field investigation was conducted as part of an emergency public health response under the authority of the Ministry of Health of the Democratic Republic of the Congo and Africa CDC. The assessment involved analysis of routine surveillance and operational data collected during outbreak response activities. No individual patient identifiers were collected or reported. Findings were used exclusively to support public health decision-making and strengthen outbreak response operations in accordance with international outbreak investigation guidelines [18].

## 3. Results

### 3.1. Epidemiological Situation

As of 12 July 2026, the outbreak continued to expand, with 53 newly confirmed cases of Bundibugyo Ebola virus disease (BDBV) and 30 deaths reported within the previous 24 h, including 20 community deaths (66.7%), indicating ongoing delays in case detection and access to care. Ituri Province remained the principal hotspot, reporting 45 new cases and 27 deaths, while North Kivu Province recorded 8 new cases and 3 deaths. Cumulatively, the outbreak reached 1926 laboratory-confirmed cases and 702 deaths, corresponding to an overall case fatality rate (CFR) of 36.4%. During the same reporting period, 10 deaths occurred among hospitalized patients receiving care in Centers for Ebola Virus Disease (EVD) Treatment (CTEs), including nine CTEs. The epidemic also expanded geographically, with Ariwara Health Zone in Ituri Province reporting its first confirmed cases, underscoring the continued risk of spread to previously unaffected areas. Table 1 provides the distributions of the cases in the different health zones/Provinces of DRC.

### 3.2. Surveillance, Contact Tracing, and Laboratory Performance

A total of 766 alerts were reported across the three affected provinces, of which 678 (88.5%) were investigated. Investigation performance was highest in North Kivu and South Kivu (100%) but remained lower in Ituri (68.1%). These investigations identified 235 suspected cases, including 61 community deaths requiring further assessment. Contact tracing remained a major weakness, with only 4171 of 6475 registered contacts successfully followed. During the reporting period, 466 new contacts were identified, most originating from Rwampara Health Zone. Laboratory activities included testing 137 specimens, with 29 positive results, yielding an overall positivity rate of 21.2%. Table 2 provides field investigations of the cases and contact tracing throughout the affected health zones of Bunia and other affected provinces.

### 3.3. Operational Response Activities and Challenges

Clinical management activities recorded 61 new admissions, 14 deaths, and one recovery during the reporting period. Treatment facilities were operating close to capacity, particularly in Ituri, where occupancy reached 97.3%. IPC activities included training of healthcare workers and decontamination of health facilities, although shortages of PPE, chlorine, and other essential supplies persisted. Community engagement activities reached thousands of households through sensitization campaigns, household visits, and engagement with political and religious leaders. Border surveillance remained active, with 44 of 55 Points of Entry and Control operational and more than 56,000 travelers screened. However, insecurity continued to undermine response efforts, including attacks against safe and dignified burial teams in Mongbwalu and the temporary detention of five frontline responders. Table 3 provides operational response strengths, challenges, and priority recommendations during the Bundibugyo Ebola virus disease (BDBV) outbreak, Democratic Republic of the Congo, July 2026.

## 4. Discussion

The 2026 Bundibugyo Ebola virus disease (BDBV) outbreak in eastern Democratic Republic of the Congo (DRC) represents the largest documented outbreak caused by *Bundibugyo ebolavirus* since its identification in Uganda in 2007. This field investigation highlights the interaction between intense transmission, fragile health systems, insecurity, population mobility, and social challenges affecting outbreak control. By July 2026, sustained transmission had resulted in substantial morbidity and mortality, with Ituri Province remaining the epicenter. Although important response capacities were established, including decentralized laboratory testing, Ebola treatment centres (CTEs), surveillance systems, and community engagement platforms, persistent gaps in contact tracing, case investigation, clinical capacity, data management, IPC implementation, and risk communication continued to limit interruption of transmission chains.

The geographic distribution of cases demonstrated substantial heterogeneity across affected provinces. Ituri Province accounted for more than 90% of confirmed cases, with transmission concentrated in Bunia, Rwampara, and Mongbwalu health zones. The high burden in Ituri likely reflects the combined effects of population displacement, artisanal mining activities, urban–rural mobility, and intense community interactions that facilitate transmission. Previous studies have shown that mobile populations associated with mining and informal economic activities are difficult to capture through conventional surveillance approaches and may contribute to silent transmission and geographic dissemination during outbreaks [7,11]. In contrast, North Kivu reported fewer cases but a higher case fatality rate, likely reflecting delayed access to care, insecurity-related barriers, reduced healthcare utilization, and differences in health-service availability. Similar provincial disparities were observed during the 2018–2020 North Kivu–Ituri Ebola outbreak, where insecurity and community mistrust affected response effectiveness [9,11].

The current outbreak shares several operational challenges with previous Ebola epidemics in DRC. During the 2018–2020 North Kivu–Ituri Ebola outbreak, difficulties in contact tracing, community resistance, attacks against response teams, and insecurity were major barriers to containment [9,11]. Similar challenges were observed during the present outbreak, demonstrating that improvements in technical capacity alone are insufficient when social and security conditions remain unfavorable. However, the current response benefited from lessons learned from previous epidemics, including more decentralized diagnostic capacity and improved outbreak management structures. Expansion of molecular testing platforms has reduced delays in confirmation compared with earlier outbreaks, although laboratory decentralization requires continued investment in quality assurance, workforce capacity, specimen transport systems, and genomic surveillance [13].

Compared with major Ebola outbreaks in other continents, particularly the 2014–2016 West Africa Ebola epidemic in Guinea, Liberia, and Sierra Leone, the current BDBV outbreak illustrates both progress and persistent vulnerabilities [20]. The West African epidemic demonstrated how delayed recognition, weak health systems, limited laboratory capacity, and insufficient community engagement can accelerate epidemic expansion [3,4,20]. In contrast, DRC entered the current outbreak with greater Ebola response experience, established treatment infrastructure, and improved diagnostic capacity. Nevertheless, common challenges remain across settings, particularly the importance of trust, community participation, culturally appropriate communication, and management of misinformation. Evidence from previous outbreaks confirms that community acceptance strongly influences adherence to isolation, contact tracing, vaccination, and safe burial interventions [14].

The findings also highlight important implications for implementation of the International Health Regulations (IHR 2005) [15]. Although DRC has strengthened several core capacities following repeated Ebola emergencies, persistent weaknesses remain in surveillance sensitivity, workforce availability, emergency coordination, laboratory networks, and risk communication. The emergence of cases in additional provinces, including Tshopo and Haut-Uélé, demonstrates the need for sustained preparedness beyond outbreak epicenters. Strengthening IHR capacities requires maintaining decentralized surveillance and diagnostic systems, improving cross-border collaboration, enhancing information sharing, and ensuring that provincial health systems can rapidly detect and respond to emerging threats.

Several strengths and limitations should be considered when interpreting these findings. The investigation benefited from the integration of multiple information sources, including epidemiological surveillance data, laboratory records, health-facility assessments, direct observations, and stakeholder interviews, allowing a broad assessment of response performance. The inclusion of operational domains such as infodemic management, community engagement, governance, and security provides additional insight into factors influencing outbreak control. However, the assessment was conducted during a rapidly evolving outbreak, and some epidemiological indicators may have changed after data collection. Incomplete outcome classification, inconsistencies between surveillance and laboratory databases, and restricted access to some areas because of insecurity may have resulted in underestimation of transmission and operational challenges.

Clinically, the findings emphasize the need to strengthen early detection, rapid referral, supportive care, and treatment capacity in high-transmission areas. The occurrence of community deaths and high occupancy of treatment centres indicates continued delays in accessing appropriate care. Expanding clinical networks, integrating peripheral health facilities into Ebola referral pathways, strengthening triage and isolation capacity, and maintaining healthcare worker training in IPC and clinical management are essential priorities. From a policy perspective, outbreak preparedness should move beyond emergency response toward sustainable health-system resilience. This includes strengthening community-based surveillance, improving data harmonization, maintaining laboratory readiness, institutionalizing risk communication and infodemic management approaches, and integrating security-sensitive planning into epidemic preparedness strategies [8,12]. The 2026 BDBV outbreak demonstrates the continuing challenges of controlling Ebola virus disease in conflict-affected and socially complex environments. While advances in laboratory decentralization, treatment infrastructure, and response coordination have improved outbreak capacity, sustained transmission reflects unresolved vulnerabilities in surveillance, contact tracing, community trust, health-system resilience, and operational access. Addressing these gaps will require coordinated action among national authorities, regional institutions, and international partners, consistent with IHR (2005) principles. The lessons from this outbreak should guide future Ebola preparedness strategies in the Great Lakes region and other settings at risk of emerging infectious disease threats.

## 5. Conclusions

The 2026 Bundibugyo Ebola virus disease outbreak in the Democratic Republic of the Congo remains a serious and ongoing public health emergency, with sustained transmission primarily concentrated in Ituri Province. The outbreak is characterized by a high burden of confirmed cases, continued mortality, and suboptimal contact follow-up, indicating ongoing gaps in interrupting transmission chains. Despite improvements in decentralized laboratory testing, key weaknesses persist in surveillance performance, contact tracing, and timely response actions. Operationally, the response is constrained by shortages of trained personnel, limited logistics and transport capacity, inadequate infection prevention and control resources, and insufficient treatment infrastructure. Insecurity and attacks on response teams further disrupt essential public health activities, while community resistance in some areas continues to hinder effective implementation of control measures. Overall, the combination of epidemiological spread, fragile health systems, and security challenges underscores a high risk of continued transmission and potential regional spread unless urgent corrective actions are sustained.

## Figures and Tables

**Table 1:** Distribution of cumulative confirmed Bundibugyo Ebola virus disease (BDBV) cases and deaths by province and health zone, Democratic Republic of the Congo, Situation Report 57 (reporting date: 10 July 2026).

Province/Health Zone	Confirmed Cases, n	% of Total Cases	Deaths, n	Crude CFR (%)	% of Total Deaths
Ituri Province	1705	90.8	577	33.8	85.5
Bunia	503	26.8	156	31.0	23.1
Rwampara	384	20.5	89	23.2	13.2
Mongbwalu	329	17.5	171	52.0	25.3
Nizi	110	5.9	36	32.7	5.3
Nyankunde	97	5.2	23	23.7	3.4
Other health zones	282	15.0	102	36.2	15.1
North Kivu Province	165	8.8	94	57.0	13.9
Katwa	67	3.6	45	67.2	6.7
Butembo	43	2.3	20	46.5	3.0
Beni	30	1.6	20	66.7	3.0
Musienene	9	0.5	2	22.2	0.3
Kyondo	5	0.3	3	60.0	0.4
Other health zones	11	0.6	4	36.4	0.6
South Kivu Province	3	0.2	1	33.3	0.1
Miti-Murhesa	3	0.2	1	33.3	0.1
Tshopo Province	3	0.2	2	66.7	0.3
Makiso-Kisangani	2	0.1	2	100.0	0.3
Mangobo	1	0.1	0	0.0	0.0
Haut-Uélé Province	1	0.1	1	100.0	0.1
Wamba	1	0.1	1	100.0	0.1
Total	1877	100.0	675	36.0	100.0

Abbreviations: BDBV, Bundibugyo Ebola virus disease; CFR, case fatality rate. Source: Ebola BDBV Situation Report 57, Democratic Republic of the Congo (reporting date: 10 July 2026; publication date: 11 July 2026).

**Table 2:** Surveillance, Contact Tracing, and Laboratory Performance Indicators.

Indicator	Ituri	North Kivu	South Kivu	Total
Alerts reported	276	483	7	766
Alerts investigated	188	483	7	678
Investigation rate (%)	68.1	100.0	100.0	88.5
Suspected cases identified	176	52	7	235
Suspected deaths identified	45	16	0	61
Contacts under follow-up	5208	1187	80	6475
Contacts successfully followed	3397	700	74	4171
New contacts listed (24 h)	466	0	0	466
Samples tested	130	NA	7	137
Positive laboratory results	29	NA	0	29
Laboratory positivity rate (%)	22.3	NA	0.0	21.2

**Table 3:** Operational response strengths, challenges, and priority recommendations during the Bundibugyo Ebola virus disease (BDBV) outbreak, Democratic Republic of the Congo, July 2026.

Response Domain	Strengths	Challenges	Priority Recommendations
Surveillance and contact tracing	Active surveillance systems remained operational with continued case detection and contact listing.	Incomplete contact listing and contact follow-up remained below the recommended ≥95% target, increasing the risk of undetected transmission. Community-based detection and reporting of alerts and deaths remained inadequate despite ongoing transmission.	Strengthen epidemiological investigations for all confirmed and probable cases; ensure comprehensive contact listing and maintain ≥95% contact follow-up; reinforce supportive supervision, active case finding, community-based surveillance, and cross-border surveillance.
Case investigation and epidemiology	Epidemiological investigations continued for confirmed and suspected cases.	Variable quality of investigations, including incomplete documentation of exposures, travel history, risk factors, epidemiological links, and transmission chains. Approximately 50 probable cases remained unclassified, while nearly 500 confirmed cases had unknown final outcomes.	Complete validation of probable cases within 72 h, actively trace confirmed cases with unknown outcomes, standardize investigation protocols, improve epidemiological documentation, and strengthen data verification and outcome reporting.
Clinical care	Functional Ebola treatment centres (CTEs) admitted 61 new patients, managed 376 patients, and maintained an overall occupancy rate of 86.8% (97.3% in Ituri).	Frequent saturation of CTEs and transit centres (>95% occupancy), insufficient treatment capacity, and continued management of patients in non-designated facilities outside the official response system.	Integrate parallel care pathways into the national response, establish additional transit centres with triage, isolation, and specimen collection capacity, accelerate the operationalization of new CTEs, and strengthen referral and clinical management systems.
Laboratory services and data management	Decentralized molecular diagnostic platforms remained operational and confirmed positive specimens during the reporting period.	Delays in specimen transport and post-mortem sampling, together with inconsistencies between surveillance, laboratory, admission, mortality, and recovery databases.	Strengthen specimen referral systems, ensure traceability of every specimen from collection to laboratory confirmation, reconcile surveillance and laboratory databases daily, and establish a unified outbreak dashboard for real-time monitoring and decision-making.
Infection prevention and control (IPC)	Healthcare workers received IPC training, and multiple health facilities underwent decontamination.	Persistent shortages of personal protective equipment (PPE), chlorine, and IPC supplies increased the risk of healthcare-associated transmission.	Ensure uninterrupted IPC supplies, strengthen supportive supervision, reinforce adherence to IPC standards, and conduct regular IPC assessments in healthcare facilities.
Risk communication, community engagement, and infodemic management	Extensive household visits and community outreach were conducted with the engagement of political, religious, and community leaders.	Community resistance, misinformation, rumors, attacks on burial teams, and low trust in public health authorities reduced acceptance of surveillance, safe burials, and treatment services.	Strengthen risk communication and community engagement (RCCE), establish systematic rumor surveillance and community feedback mechanisms, engage trusted local leaders, implement culturally appropriate communication strategies, and rapidly identify and address misinformation to improve trust and uptake of public health interventions.
Points of Entry (PoE) and Points of Control (PoC)	Most PoE/PoC sites remained operational with high traveller screening coverage.	Continued population mobility and non-operational screening sites increased the risk of cross-border spread.	Restore full functionality of all PoE/PoC sites, strengthen cross-border coordination and information sharing, and maintain comprehensive traveller screening, referral, and follow-up.
Geographic expansion and preparedness	Surveillance and response activities expanded into newly affected areas.	Ongoing spread to Tshopo, Haut-Uélé, and other high-risk provinces highlighted insufficient preparedness and response capacity.	Pre-position multidisciplinary response teams, diagnostic capacity, essential supplies, transit centres, and coordination mechanisms in high-risk provinces to improve preparedness and rapid response.
Human resources, coordination, and governance	Multidisciplinary response teams remained operational despite a challenging security environment.	Workforce constraints included inconsistent remuneration policies, delayed payments, staff strikes, operational delays associated with quarantine measures, and unclear governance structures with overlapping responsibilities.	Harmonize human resource management policies, ensure timely deployment and rotation of response personnel using risk-based quarantine approaches, clarify roles and accountability of Incident Management Team leadership, strengthen coordination across response pillars, and institute routine performance reviews at national and subnational levels.
Security and operational access	Response activities continued despite insecurity in affected areas.	Attacks on burial teams, detention of response workers, and insecurity restricted access to affected communities and disrupted response operations.	Strengthen security risk assessments, coordinate closely with local authorities and community leaders, implement context-specific security measures, and ensure safe and sustained access for frontline response teams.

## Data Availability

All data supporting the findings of this study are included in the article and its tables. Additional aggregated epidemiological and operational datasets may be available from the corresponding author upon reasonable request and with permission from the Ministry of Health of the Democratic Republic of the Congo.
